# Cross-sectional and longitudinal associations between parenting style and adolescent girls’ physical activity

**DOI:** 10.1186/1479-5868-9-141

**Published:** 2012-12-03

**Authors:** Julie Saunders, Clare Hume, Anna Timperio, Jo Salmon

**Affiliations:** 1 School of Population Health, University of Western Australia, Perth, Western Australia, 6009; 2 School of Exercise and Nutrition Sciences, Deakin University, Burwood, Victoria, 3125, Australia

**Keywords:** Parental influences, Family environment, Adolescents’ Physical activity

## Abstract

**Background:**

Understanding the influences on physical activity is crucial, particularly among important target groups such as adolescent girls. This study describes cross-sectional and longitudinal associations between parenting style and girls’ participation in organized sport, walking/cycling trips and objectively assessed moderate to vigorous physical activity (MVPA).

**Methods:**

Data were collected from adolescent girls (n=222) and their parents in 2004 and again in 2006. Parents self-reported their demographic characteristics and parenting style. Girls self-reported their organized sport participation and weekly walking/cycling trips, while MVPA was assessed using accelerometers. Linear regression and interaction analyses were performed. Interactions between socio-demographic factors and parenting style with organized sport, walking/cycling trips and MVPA are presented.

**Results:**

There were cross-sectional associations between authoritative (B=−0.45, p=0.042) and indulgent (B=−0.56, p=0.002) parenting and the number of walking/cycling trips, and authoritarian (B=0.27, p=0.033) parenting and frequency of organized sport. Significant interactions included those between: family status, authoritative parenting and daily (p=0.048) and week day (p=0.013) MVPA; education, indulgent parenting and MVPA on weekend days (p=0.006); and, employment, authoritarian parenting and duration and frequency of organized sport (p=0.004), highlighting the complexity of these relationships. Longitudinal analyses revealed significant decreases in organized sport and MVPA, significant increases in walking/cycling trips and no significant associations between parenting and physical activity.

**Conclusion:**

Parenting styles appear to influence walking and cycling trips among adolescent girls, though not physical activity within other domains. Socio-demographic characteristics interact with the relationships between parenting and physical activity. While these findings can inform the development of family-based interventions to improve child and adolescent health, the direction of the observed associations and the number of associations approaching significance suggest the need to further explore this area.

## Background

Among adults, the association between regular physical activity and reductions in morbidity and mortality is well established [1]. Whilst the body of research into the benefits of physical activity among children is not as extensive, there is growing support for the role of physical activity in bone health and emotional well-being [2], reduction in coronary heart disease (CHD) risk factors [3] and social and moral development and self esteem [4].

The transition from childhood to adolescence has been identified as a period of marked decline in physical activity [5-7], particularly amongst girls [4]. Indeed, girls appear to be less physically active than boys across all age groups [8]. Sex differences in the types and intensities of physical activity engaged in have also been reported, with boys undertaking more vigorous-intensity physical activity (VPA) [9], moderate- to vigorous-intensity physical activity (MVPA) [10], organized sport [11] and walking and cycling in the local neighbourhood [12] than girls. Understanding the influences on participation among girls is necessary to increase physical activity among this important target group.

It is useful to consider potential influences on behaviour under the guidance of theory. The Family Influence Model (FIM) [13,14] purports that the home environment (consisting of parent/sibling beliefs, parent/sibling behaviour, and family functioning and interaction) influences a child’s perception of the home environment. This perception then leads to the development of specific beliefs which in turn is a primary influence on behaviour [14]. In a physical activity context, the FIM has been used to explain the influence of the family environment on children’s MVPA [13], and posits that parents’ beliefs about their children’s MVPA is the basis for understanding family influence on children’s MVPA.

Constructs within the FIM, including factors within the proximal family environment, such as parent support, support from significant others, sibling physical activity and direct help from parents, have been consistently associated with adolescents’ physical activity [8]. Parents’ provision of logistic support and explicit modelling has been associated with girls’ physical activity [15,16], while among female adolescents, exercise knowledge and mothers’ modelling/support [17] have been identified as correlates of physical activity participation. Similarly, among inactive adolescent girls, support for physical activity from parents was a strong and consistent correlate of physical activity participation [18]. Other constructs within the FIM, such as parents’ behaviour and family processes, have not been fully tested and family characteristics such as parenting style could be examined within this framework. Further, this model acknowledges the influence of socio-demographic factors on physical activity and the home environment [14], making it a useful tool for examining potential interactions between socio-demographic factors, parenting and physical activity.

Parenting style is a stable characteristic within the family environment [19], which has been associated with various health outcomes among adolescents [20,21]. The literature identifies four main parenting styles, which are reflective of various degrees of demandingness (control) and responsiveness (support) [20-22]. Authoritative parents are considered responsive, nurturing, set clear expectations and explain the reasons behind these expectations [23]. Authoritarian parents are firm and directive, relatively unresponsive, value obedience and exclude the child from decision making [20,23]. Indulgent parents place few demands on the child and are child-oriented, responsive and nurturing, while neglectful parents provide relatively low support and control [20,23]. Recent research suggests that authoritative feeding practices are associated with child consumption of fruit and vegetables [24,25] and authoritarian parenting with risk of overweight among young children [19], although a recent review notes the lack of causal evidence [26]. While preliminary data demonstrate an association between authoritative parenting and girls’ physical activity [27], few studies have comprehensively examined how parenting style influences physical activity, despite such studies being recognised as imperative [28]. Further, the need for more longitudinal research in the area, employing a combination of self-report and objective measures, has been identified [16].

Given the important influence of parenting style on child and adolescent health behaviours and health, and the known associations between other aspects of the family environment (such as provision of support and direct help from parents) and physical activity, it is plausible that parenting style may influence adolescent physical activity. Further, socio-demographic characteristics previously associated with physical activity, such as educational attainment, may interact with parenting style to influence physical activity. The present study describes cross-sectional and longitudinal associations between parenting style and adolescent girls’ participation in organized sport, walking/cycling trips and objectively assessed MVPA and explores potential interaction with socio-demographic factors.

## Methods

These analyses are based on a sub-sample (adolescent girls) from the Children Living in Active Neighbourhoods (CLAN) cohort study. The study combined questionnaire and accelerometry data to examine contextual influences on physical activity. Ethics approval was obtained from Deakin University Ethics Committee and permissions were received from the Department of Education and Training Victoria and the Catholic Education Office. Informed written consent was received from parents and written assent from adolescents.

### Sample

In 2001, 919 10–12 year old children (n=495 girls) and their parents were recruited through 19 primary schools in high and low SES areas in Melbourne, Victoria. Details on baseline recruitment and sample selection are described elsewhere [29]. In 2004, 222 adolescent girls and their parents participated in a 3-year follow-up. Data were collected between July and December 2004. In 2006, 166 adolescent girls and/or their parents participated in a second follow up during the same months. Measures of parenting style were only collected in 2004, thus data from 2004 are considered baseline and 2006 considered follow-up for the purposes of this paper. Physical activity and socio-demographic data were collected in both 2004 and 2006. Although boys and younger children also participated in the CLAN study, this paper includes only adolescent girls as they are at particularly high risk for physical inactivity.

### Measures

#### Survey measures

Parents or carers completed survey items regarding socio-demographics and parenting style and adolescents completed survey items relating to organized sport participation and walking and cycling to school.

##### Socio-demographic items

Parents/carers reported their relationship to the child in the study and their age, educational attainment (collapsed into some secondary school or less (low); completed secondary school, technical college or apprenticeship (mid); university/tertiary qualification (high)) and employment status (collapsed into employed full-time; employed part-time; home duties full-time/other). Family status was recorded as dual carer if the responding parent/carer also answered the above questions about their co-carer who lives with them, and those parents who did not record responses to these questions were identified as single carer. Although marital status was also assessed, the number of carers present in the home was considered to be more likely to influence parenting style and was used in all analyses.

##### Weight status

Children’s height and weight were measured at school in a private room using digital scales and a portable stadiometer. Weight status was calculated and children defined as normal weight, overweight or obese based on international age and gender-specific cutpoints [30].

##### Parenting style

Twenty-two items assessed parenting style, for example “I make decisions in consultation with my child”, “I am clear about my parental role” and “I have the final say with my child”. Response options on a five-point scale were: never (1); rarely (2); sometimes (3); often (4); and always (5). These items were adapted from those developed by Baumrind [31]. Adaptations included simplifying the wording and developing additional items based on the constructs assessed by Baumrind. Factor analyses were used to reduce items into categories of parenting style. With the exception of three items that were reflective of specific parenting practices rather than overall parenting styles (I become annoyed/impatient when my child disobeys me; I become irritated/annoyed when my child dawdles or is annoying; I avoid open confrontation with my child), all remaining items loaded onto one of four factors/categories with Eigenvalues >1 (Table 1). These factors reflected the indulgent, authoritarian, authoritative and neglectful parenting styles identified in the literature [20-22]. The internal reliability (Cronbach’s alpha) of the parenting styles ranged from 0.62 for a neglectful parenting style to 0.77 for an authoritarian parenting style. Responses to each item within each category were summed then averaged, and the average scores dichotomised at the mean.

**Table 1:** Description of factors arising from factor analysis

**Items**	***Factors***			
	**Indulgent**	**Authoritative**	**Authoritarian**	**Neglectful**
I let my child express feelings about being punished or restricted	**.735**			
I listen to reasons why my child might not want to do something that I ask him/her to do	**.731**			
I encourage my child to tell me what he/she is thinking	**.692**			
I make decisions in consultation with my child	**.611**			
I tell my child how happy he/she makes me	**.491**			
I am consistent with my discipline techniques		**.753**		
I make clear rules for my child to follow		**.706**		
I give my child reasons for my directions		**.641**		
I am clear about my parental role		**.537**		
I use a gentle manner with my child		**.441**		
I confront my child when he/she does not do as I say			**.738**	
I punish my child for disobedience			**.728**	
I am firm with my child			**.703**	
I have the final say with my child			**.691**	
I see to it that my child does what he/she is told			**.542**	
I let myself be talked out of things by my child				**.763**
I ignore my child’s misbehaviour				**.644**
My child nags me into changing my mind				**.625**
My child wins arguments with me				**.606**
***Eigenvalue***	***4.66***	***3.06***	***1.84***	***1.22***
% variance	21.2	13.9	8.4	5.5
Mean score for each parenting style (SD)*	3.98 (0.55)	4.08 (0.46)	3.58 (0.57)	2.39 (0.53)

* Range is from 1–5, with higher values representing greater presentation of these characteristics.

##### Organized sport participation

Participation in organized sport was self-reported using an adaptation of the Adolescent Physical Activity Recall Questionnaire (APARQ) [32], which asked the adolescent to list each organized physical activity they were involved in during summer and winter respectively, the average number of times per week they participated, and the average duration each time they participated. Responses were cleaned and truncated consistent with procedures used by Booth and colleagues [32]. Total frequency and duration of organized sport participation in summer and winter were computed for each participant, and the average frequency and duration of organized sport across the whole year was calculated. The reliability and validity of the APARQ has previously been reported as acceptable [32].

##### Walking and cycling trips

Girls were asked to report how frequently they walked or cycled to each of 15 common destinations (e.g. friends’ houses, sport venues, school and parks) in a usual week [33]. Response options (and assigned scores) were: it’s not within walking/riding distance (0); never/rarely (0); less than once/week (0); 1–2 times/week (1); 3–4 times/week (3); 5–6 times/week (5); and daily (7). Responses were summed to compute weekly frequency of walking/cycling trips. The measure has acceptable reliability [33].

#### Accelerometry

##### Moderate-to vigorous-physical activity (MVPA)

MVPA was assessed using accelerometers (Actigraph Model GT1M) [34]. The accelerometers were initialised to collect data in one minute epochs and participants were requested to wear their accelerometer on their right hip for eight consecutive days, only removing it for aquatic activities, bathing and sleeping.

Due to fitting of the accelerometer, data from day 1 was removed as it represented incomplete data. Wear-time was calculated as 24 hours minus all periods with >20 minutes of consecutive zeros. Days where wear-time was >= 8 hours and <300 minutes of vigorous activity was recorded were included as valid days. Total counts per minute were converted into duration of movement at various intensities according to the age-specific cutpoints utilised by Freedson and colleagues [35], using a custom-designed data reduction program. Moderate-intensity activity was defined as 4.0-5.9 METs and vigorous-intensity as 6.0 METs and above [34]. Minutes per day in MVPA were calculated by summing the minutes spent in moderate activity and the minutes spent in vigorous activity. Average duration of MVPA on weekdays, weekend days, and across the week was calculated. MVPA recorded during the ‘critical window’ or after school period from 3pm to 6pm, was also calculated.

Participants were required to have 4 or more valid days (including 1 or more weekend day) of data for inclusion in weekly MVPA analyses, 3 or more valid weekdays for inclusion in weekday analyses, 1 or more valid weekend day for inclusion in weekend analyses and 3 or more valid days for inclusion in critical window analyses.

### Data transformation

In both 2004 and 2006, organized sport, walking/cycling trips and MVPA data were all positively skewed and were therefore transformed, with the square root transformation best approximating a normal distribution for all physical activity variables. Transformed data were used for all statistical analyses and generation of p-values. Unless specified otherwise, transformed data have been reported in tables, with corresponding raw values described in text.

### Statistical analyses

Data were managed and analysed using IBM SPSS Statistics Version 19 (2010). Descriptive statistics were used to describe demographic characteristics. Regressions were performed to determine associations between socio-demographics and physical activity at baseline. Separate bivariable linear regression models were generated to assess associations between independent (parenting style) and dependent (organized sport, MVPA and walking/cycling trips respectively) variables. General Linear Modelling (GLM) was employed to examine interactions between 1) specific socio-demographic variables (parental employment, parental education and family status) and parenting style and organized sport; 2) specific socio-demographic variables and parenting style and walking/cycling trips; and 3) specific socio-demographic variables and parenting style and MVPA.

Paired t-tests were used to describe changes in physical activity from 2004 to 2006 and bivariable linear regressions were performed to examine associations between parenting style in 2004 and physical activity in 2006, controlling for baseline physical activity and, where appropriate, socio-demographics.

## Results

### Demographic characteristics

In 2004, the mean age of the girls (n=222) in the sample was 14.5 (SD 0.6) years. Most were not overweight or obese, with 74% of girls classified as within the normal weight range. The mean age of the responding parent was 43.9 (SD 5.1) years and the majority were mothers (87%), employed either full time or part time (77%) and were part of a dual carer family (80%). Almost half (44%) had completed a university or tertiary qualification. In 2006 (follow-up), the mean age of girls (n=166) was 16.3 (SD 0.6) years and most (73%) were within the normal weight range. The mean age of the responding parent was 46.2 years (SD 4.8) and again most were mothers (86%), employed either full time or part time (82%), part of a dual carer family (80%) and university or tertiary educated (48%).

There were no significant differences in any of the variables examined between the 166 girls who were retained in the sample from 2004 to 2006 and those who were lost to follow up (n=56). Therefore, to maximise the baseline sample size, cross-sectional analyses were performed using all available data rather than restricting the sample to only those who also participated in 2006.

### Physical activity participation

Participation in organized sport, number of walking/cycling trips/week and duration of MVPA in 2004 and 2006 is presented in Table 2. Significant decreases in all organized sport and MVPA variables were observed, while the number of weekly walking/cycling trips increased significantly.

**Table 2:** Organized sport, walking/cycling trips and MVPA participation in 2004 and 2006

	**Cross-sectional**		**Longitudinal**			
	**Mean (SD)**		**2004Mean (SD)**		**2006Mean (SD)**	p-value*
**Organized sport**		**N**		**N**		
Frequency (times/week)	4.5 (4.1)	203	4.4 (3.8)	160	3.3 (3.4)	0.001
Duration (hrs & mins/week)	5h06m (4h30m)	202	5h01m (4h19m)	159	3h56m (3h45m)	0.006
**Walking/cycling trips** Trips per week	6.8 (7.3)	222	7.3 (7.6)	166	10.6 (7.9)	0.000
**MVPA (mins/period)**						
Average day	38.3 (18.1)	140	39.4 (18.2)	85	23.8 (15.8)	0.000
Weekdays	42.2 (20.3)	152	44.8 (20.6)	97	27.4 (18.5)	0.000
Weekend days	26.1 (34.0)	125	24.7 (29.5)	68	15.8 (22.7)	0.011
Critical window (3-6pm)	13.1 (9.9)	148	14.1 (10.7)	96	9.0 (8.8)	0.000

*Paired samples t-tests (based on transformed data); untransformed data reported in table.

### Associations between socio-demographics and physical activity

There were no associations between parent employment status or parental education, and any of the organized sport, walking/cycling trips or MVPA variables in 2004. Family status was associated with walking/cycling trips (p=0.002), but not with organized sport or MVPA. Where applicable, these variables were controlled for in cross-sectional and longitudinal analyses.

### Cross-sectional associations between parenting style and physical activity

Cross-sectionally, an authoritarian parenting style was positively associated with frequency of organized sport participation (p=0.033), with each unit increase in authoritarian parenting resulting in 1.1 additional instances of organized sport participation per week. The number of walking/cycling trips per week was negatively associated with authoritative (p=0.042) and indulgent (p=0.002) parenting, with each unit increase in authoritative parenting resulting in 2.0 fewer walking/cycling trips per week and each unit increase in indulgent parenting resulting in 2.9 fewer walking/cycling trips per week. There was a trend towards an increased duration of organized sport with authoritarian parenting, although this finding was not statistically significant. There were no statistically significant associations between MVPA on average days, weekdays, weekend days or the after school period and parenting style.

In multivariable regression analyses, an indulgent parenting style was significantly inversely associated with walking/cycling trips (B= −2.83; 95% CI:-4.80, -0.86, p=0.005) (Table 3).

**Table 3:** Bivariate associations between parenting style and organized sport, walking/cycling trips and MVPA in 2004

	**Org sport frequency/wk**	**Org sport duration/wk**	**Number of walking/cycling trips/ wk**^**#**^	**Mins MVPA average day**^**†**^	**Mins MVPA weekdays**^**†**^	**Mins MVPA weekend days**^**†**^	**Mins MVPA after school period**^**†**^
	**Unstandardised B (95%CI) (n=203)**	**Unstandardised B (95%CI) (n=202)**	**Unstandardised B (95%CI) (n=222)**	**Unstandardised B (95%CI) (n=140)**	**Unstandardised B (95%CI) (n=152)**	**Unstandardised B (95%CI) (n=125)**	**Unstandardised B (95%CI) (n=148)**

**Parenting style**							
Authoritarian	0.27 (0.02, 0.52)*	1.94 (−0.14, 4.03)	−0.05 (−0.40, 0.31)	0.07 (−0.36, 0.50)	−0.27 (−0.69, 0.15)	0.50 (−0.33, 1.34)	−0.18 (−0.53, 0.16)
Authoritative	0.04 (−0.28, 0.36)	0.62 (−2.04, 3.28)	−0.45 (−0.88, -0.02)*	0.03 (−0.51, 0.56)	−0.08 (−0.61, 0.46)	0.21 (−0.83, 1.25)	0.00 (−0.44, 0.44)
Indulgent	0.15 (−0.12, 0.41)	1.26 (−0.94, 3.46)	−0.56 (−0.92, -0.20)**	−0.17 (−0.62, 0.27)	−0.41 (−0.85, 0.03)	0.44 (−0.43, 1.31)	−0.20 (−0.56, 0.17)
Neglectful	−0.21 (−0.48, 0.07)	−1.35 (−3.64, 0.94)	0.16 (−0.22, 0.54)	−0.05 (−0.51, 0.42)	0.09 (−0.56, 0.37)	0.29 (−0.61, 1.19)	0.11 (−0.27, 0.48)

^*#*^*controlling for family status;*^†^MVPA analyses adjusted for wear time; *p<=0.05; **p<0.01; transformed data reported in table

### Interactions between parenting style, socio-demographics and physical activity

**Figure 1:**
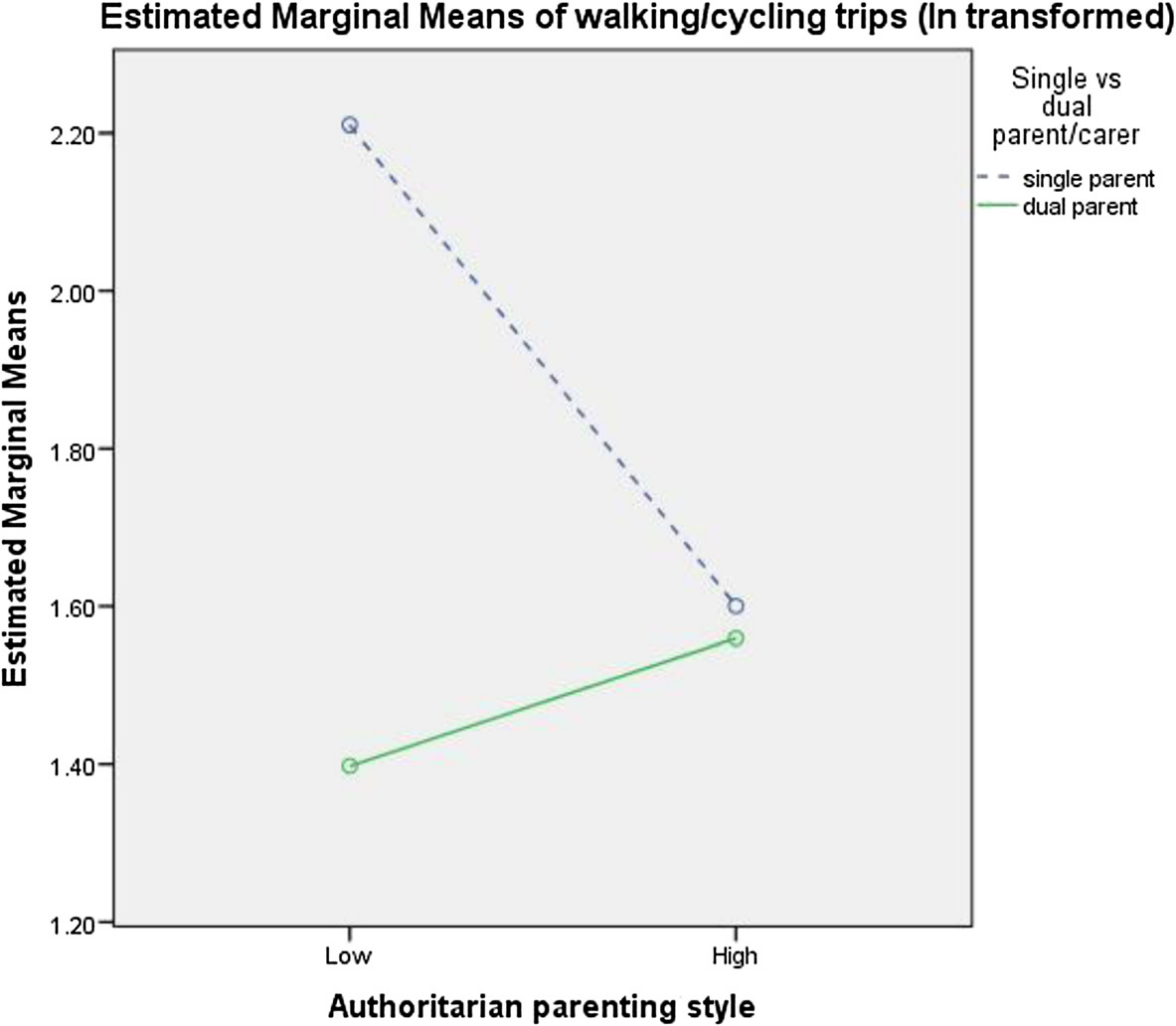
Interaction between family status, authoritarian parenting and walking/cycling trips.

**Figure 2:**
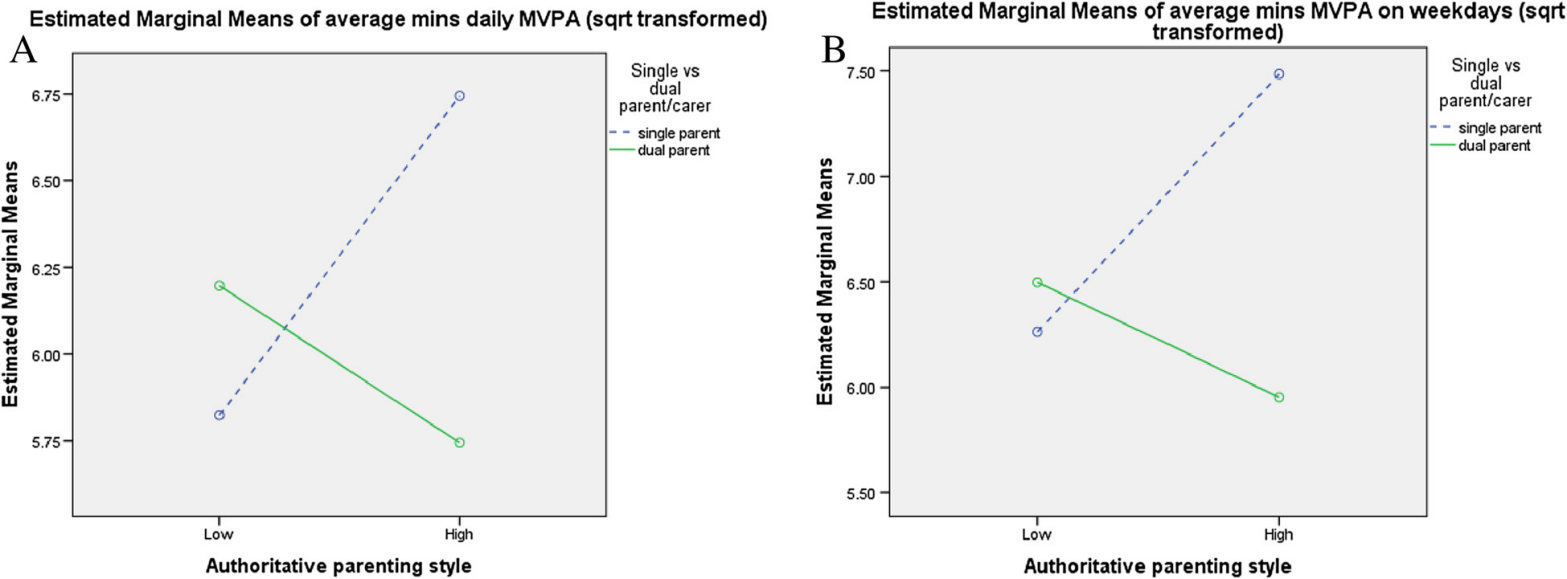
Interaction between family status, authoritative parenting and a) daily MVPA and b) MVPA on weekdays.

**Figure 3:**
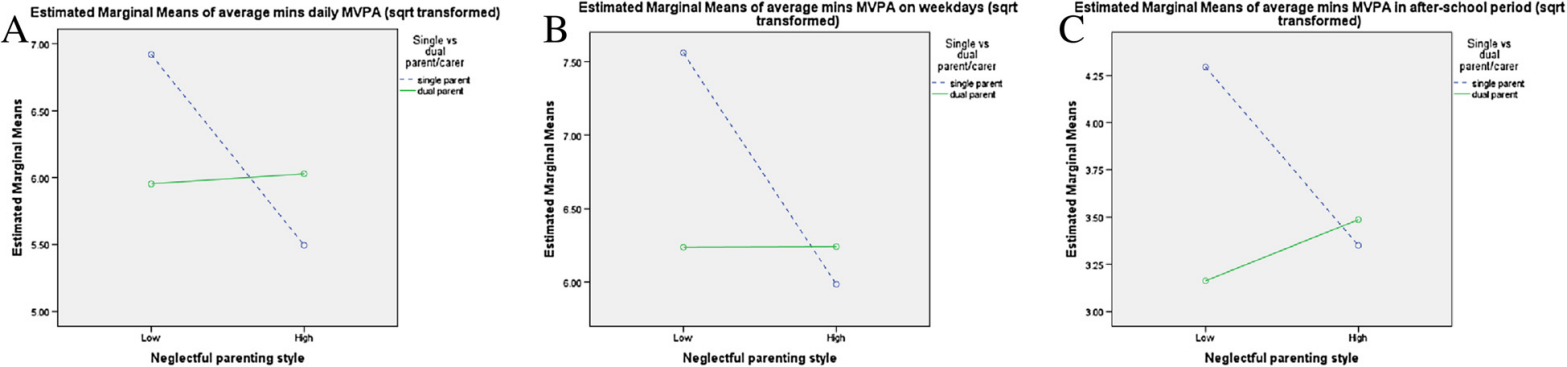
Interaction between family status, neglectful parenting and a) daily MVPA, b) weekday MVPA and c) MVPA in the after school period.

**Figure 4:**
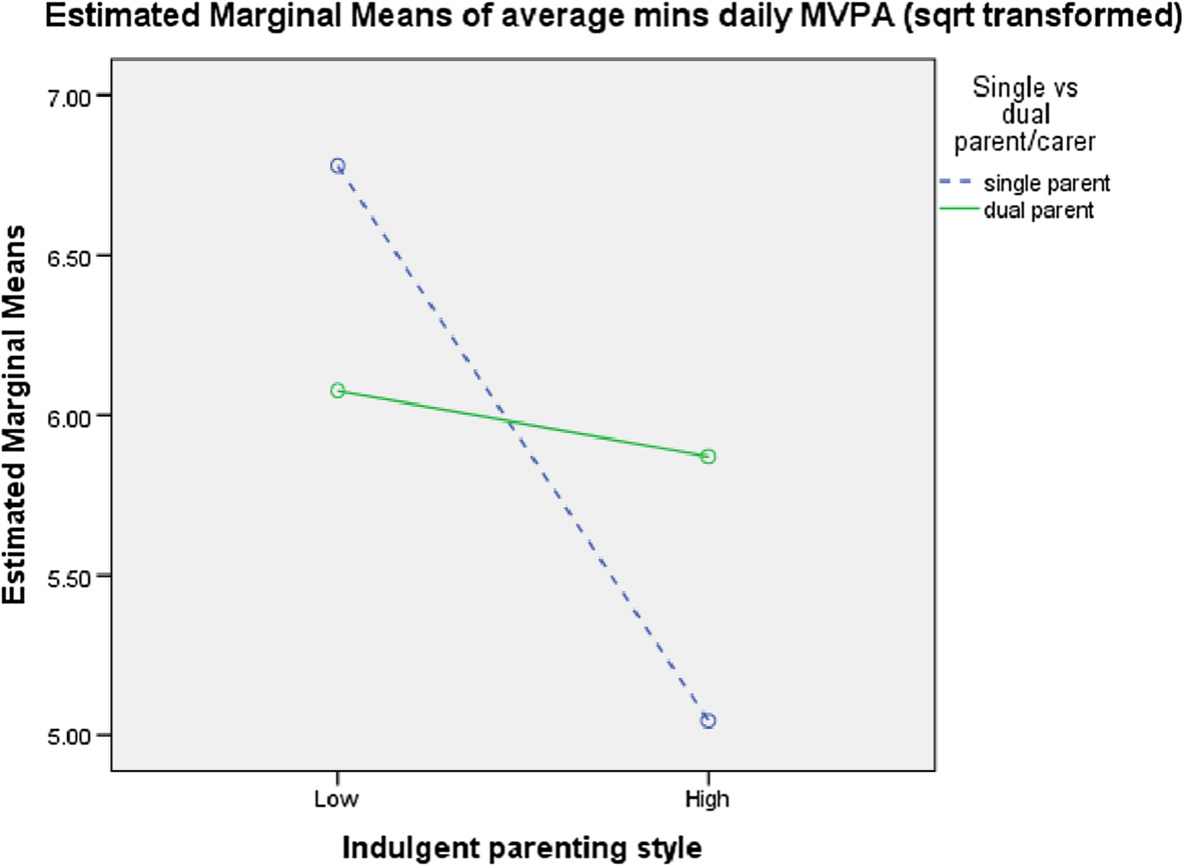
Interaction between family status, indulgent parenting and daily MVPA.

**Figure 5:**
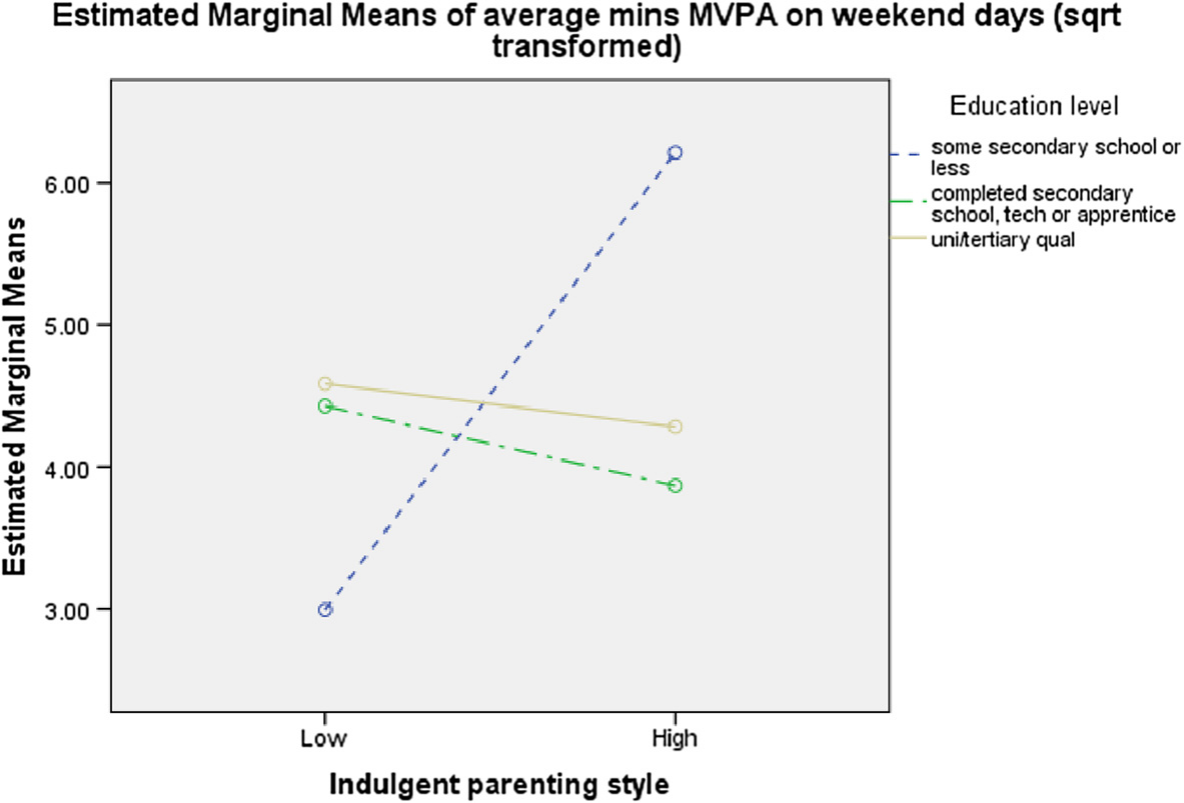
Interaction between parental education, indulgent parenting and MVPA on weekend days.

**Figure 6:**
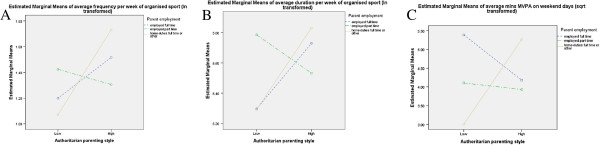
Interaction between parental employment status, authoritarian parenting and a) organized sport frequency, b) organized sport duration and c) MVPA on weekend days.

A number of significant interactions were found between socio-demographics, parenting styles and physical activity in 2004 (Figures 1,2,3,4,5,6). A significant interaction was found between family status and an authoritarian parenting style with walking/cycling trips (F=4.378, p=0.038), with children of single carers who were less authoritarian participating in more walking/cycling trips per week than other children (Figure 1). Children of single carers who were more authoritative participated in more daily MVPA (F=3.988, p=0.048) (Figure 2a) and weekday MVPA (F=6.265, p=0.013) (Figure 2b) than other children, while children of single carers who were less neglectful participated in more daily MVPA (F=5.059, p=0.026) (Figure 3a), more weekday MVPA (F=5.236, p=0.024) (Figure 3b) and more MVPA in the after school period (F=5.196, p=0.024) (Figure 3c) than other children. Children of single carers who were more indulgent participated in less daily MVPA than their counterparts (F=5.009, p=0.027) (Figure 4).

Children of responding carers who had completed some secondary school and were more indulgent participated in more MVPA on weekend days than other children (F=5.427, p=0.006) (Figure 5), while children of responding carers who were at home full time and were less authoritarian participated in a shorter duration (F=4.606, p=0.011) (Figure 6a) and lower frequency (F=5.664, p=0.004) (Figure 6b) of organized sport each week and less weekend PA than their counterparts (F=4.061, p=0.020) (Figure 6c).

### Longitudinal associations between parenting style and physical activity

There were no significant longitudinal associations between parenting style in 2004 and physical activity variables in 2006, although a number of associations approached significance. These included an authoritative parenting style and walking/cycling trips (p=0.097) and MVPA in the after school period (p=0.071), and a neglectful parenting style and frequency (p=0.051) and duration (p=0.054) of organized sport.

## Discussion

This study aimed to explore cross-sectional and longitudinal associations between parenting style and adolescent girls’ participation in organized sport, walking/cycling trips and objectively assessed MVPA, with several associations identified as well as interactions with socio-demographic factors. Cross-sectional analyses showed associations between authoritative and indulgent parenting and walking/cycling trips, and authoritarian parenting and frequency of organized sport. Significant interactions included those between: family status, authoritative parenting and daily and week day MVPA; education, indulgent parenting and MVPA on weekend days; and, employment, authoritarian parenting and duration and frequency of organized sport, highlighting the importance of tailoring public health interventions to specific socio-demographic groups. Longitudinal analyses revealed significant decreases in organized sport and MVPA and significant increases in walking/cycling between 2004 and 2006. There were no significant longitudinal associations between parenting and physical activity. This study is one of the first to examine how parenting styles influence physical activity in a longitudinal sample.

There is very little parenting research specific to physical activity with which to compare the results of the current study, although authoritative parenting has previously been positively associated with a number of child and adolescent health outcomes [20]. The current study found a negative cross-sectional association between authoritative parenting and walking/cycling trips. This negative association may reflect authoritative parents’ provision of higher levels of support for their child, which may manifest itself in non-active transport options. Alternatively, children of authoritative parents may avail themselves of parental support by requesting parents drive them by car to neighborhood destinations. Further exploring the reasons for this finding may provide an interesting focus for future research.

The negative cross-sectional association between indulgent parenting and weekly walking/cycling trips may be explained by indulgent parents’ provision of higher levels of support for their child in the form of motorized transport, thereby reducing the need for their child to use more active transport options. In this study, each unit increase in indulgent parenting resulted in almost three fewer walking/cycling trips per week for adolescent girls, which may represent a substantial amount of activity [36]. Investigating the nuances of this relationship may therefore be important.

A positive cross-sectional association between authoritarian parenting and organized sport frequency was observed in the current study, while the positive association between authoritarian parenting and organized sport duration approached significance. It is possible this finding may be related to authoritarian parents’ placement of demands on their child, strict enforcement of rules and monitoring of behavior [20], which may be applied to their daughter’s participation in organized sport. Again, exploring this notion qualitatively may be appropriate.

The observed interactions between socio-demographics, parenting style and physical activity suggest a complex relationship between these variables and provide direction for further research and intervention, in particular for the identification of practices which are supportive of physical activity within parenting styles and in light of personal socio-demographic circumstances. For example, single parents who exhibit low levels of authoritarian parenting may provide useful insights into encouraging walking/cycling trips, while more authoritarian parents who work part-time may benefit from guidance or strategies to include organized sport in their children’s routine. Further exploration of the specific physical activity related parenting practices employed within each of the parenting styles and socio-demographic sub-groups is required.

The decrease in physical activity over the two years of this study, particularly in organized sport and MVPA, is consistent with previous studies [37-39]. The consistent evidence of declines among girls provides substantial justification for the need to address physical activity among this target group. The observed increase in active transport over the two years is also consistent with the literature [40], however further longitudinal studies are required [41]. Given the contribution that active transport appears to make to achievement of physical activity guidelines [36], it is important to ensure that parents are supportive of active transport behaviours.

There are a number of limitations to this study, including the use of global measures of parenting style that were not specific to physical activity, the relatively small sample size and the narrow age range of participants. Physical activity specific measures of parenting styles and practices should be developed and tested within larger, more representative samples. Although parenting style is a stable characteristic established early in life [19], the practices implemented within these parenting styles may evolve as children age. Investigating the influence of physical activity-related parenting practices in other age groups may be warranted. Further, a number of participants (n=56) were lost to follow-up (although there were no significant differences between those who were and were not retained in the sample on any of the key variables). The inclusion of cross-sectional and longitudinal data, providing a more comprehensive picture of the temporal relationship between parenting and physical activity, and the use of objective measures of physical activity are methodological strengths.

## Conclusions

This study provides unique data on the influence of parenting styles on physical activity, and the interactions socio-demographics characteristics have with these relationships. While few associations between parenting style and physical activity were observed, the direction of the associations and the number of associations approaching significance (data not shown) suggests the need to further explore this area. In order to better understand the potential influence of parenting on girls’ physical activity, the development of measures of parenting styles and practices specific to physical activity is required. Further, given the significant decline in physical activity participation during the transition from childhood to adolescence, investigating these associations in girls before they reach adolescence is critical.

## Competing interests

The authors declare they have no competing interests.

## Author’s contributions

All authors contributed equally to this manuscript and read and approved the final manuscript.
